# Crystal structure of (*E*)-4-ethyl-2-(4-meth­oxy­benzyl­idene)-3,4-di­hydro­naphthalen-1(2*H*)-one

**DOI:** 10.1107/S205698901501004X

**Published:** 2015-05-30

**Authors:** Mohamed Akhazzane, Ghali Al Houari, Mohamed El Yazidi, Mohamed Saadi, Lahcen El Ammari

**Affiliations:** a Laboratoire de Chimie Organique, Faculté des Sciences Dhar el Mahraz, Université Sidi Mohammed Ben Abdellah, Fès, Morocco; b Laboratoire de Chimie du Solide Appliquée, Faculté des Sciences, Université Mohammed V, Avenue Ibn Battouta, BP 1014, Rabat, Morocco

**Keywords:** crystal structure, benzyl­idene, naphthalenone, dipolar 1,3-cyclo­addition reactions

## Abstract

In the title compound, C_20_H_20_O_2_, the exocyclic C=C double bond has an *E* conformation. The ethyl substituent on the cyclo­hexa­none ring is in an axial orientation. The cyclo­hexa­none ring adopts a screw-boat conformation, with the methyl­ene C atom and the C atom bearing the 4-meth­oxy­benzyl­idene group displaced from the other atoms by 0.812 (1) and 0.334 (1) Å, respectively. The dihedral angle between the planes of the benzene rings is 42.20 (8)°. In the crystal, no directional inter­actions beyond van der Waals contacts are observed.

## Related literature   

For general background to dipolar 1,3-cyclo­addition reactions, see: Bennani *et al.* (2007 ▸); Kerbal *et al.* (1988 ▸); Al Houari *et al.* (2008 ▸). For a related structure, see: Akhazzane *et al.* (2010 ▸).
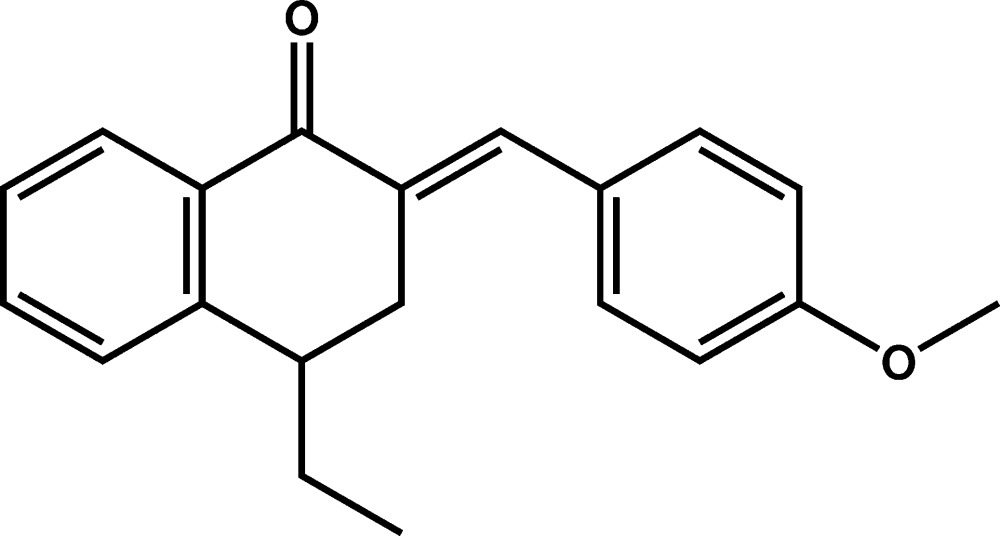


## Experimental   

### Crystal data  

C_20_H_20_O_2_
*M* *_r_* = 292.36
Monoclinic, 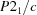

*a* = 12.0411 (13) Å
*b* = 8.9698 (9) Å
*c* = 15.5832 (18) Å
β = 110.721 (3)°
*V* = 1574.2 (3) Å^3^
*Z* = 4
Mo *K*α radiation
μ = 0.08 mm^−1^
*T* = 296 K
0.38 × 0.16 × 0.12 mm

### Data collection  

Bruker X8 APEX CCD diffractometer
25378 measured reflections
4068 independent reflections
2552 reflections with *I* > 2σ(*I*)
*R*_int_ = 0.046

### Refinement  

*R*[*F* ^2^ > 2σ(*F* ^2^)] = 0.046
*wR*(*F* ^2^) = 0.129
*S* = 1.01
4068 reflections
200 parameters
H-atom parameters constrained
Δρ_max_ = 0.16 e Å^−3^
Δρ_min_ = −0.15 e Å^−3^

### 

Data collection: *APEX2* (Bruker, 2009 ▸); cell refinement: *SAINT* (Bruker, 2009 ▸); data reduction: *SAINT*; program(s) used to solve structure: *SHELXS2013* (Sheldrick, 2008 ▸); program(s) used to refine structure: *SHELXL2013* (Sheldrick, 2015 ▸); molecular graphics: *ORTEPIII* (Burnett & Johnson, 1996 ▸) and *ORTEP-3 for Windows* (Farrugia, 2012 ▸); software used to prepare material for publication: *PLATON* (Spek, 2009 ▸) and *publCIF* (Westrip, 2010 ▸).

## Supplementary Material

Crystal structure: contains datablock(s) I. DOI: 10.1107/S205698901501004X/hb7434sup1.cif

Structure factors: contains datablock(s) I. DOI: 10.1107/S205698901501004X/hb7434Isup2.hkl

Supporting information file. DOI: 10.1107/S205698901501004X/hb7434Isup3.cml

**Fig. 1.**
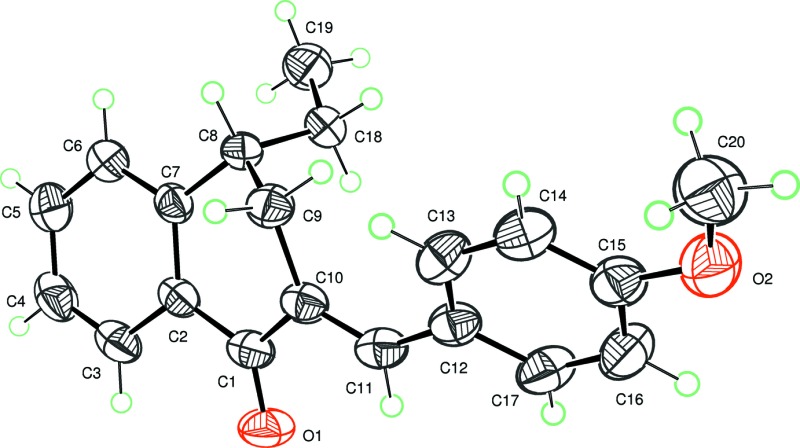
: Plot of the molecule of the title compound with displacement ellipsoids drawn at the 50% probability level.

. DOI: 10.1107/S205698901501004X/hb7434fig1.tif
Plot of the mol­ecule of the title compound with displacement ellipsoids drawn at the 50% probability level.

CCDC reference: 1402624

Additional supporting information:  crystallographic information; 3D view; checkCIF report

## supplementary crystallographic information

### S1. Comment

Knowledge of the configuration and conformation of the title compound is necessary to understand its behaviour in dipolar-1,3 cycloaddition reactions (Bennani *et al.*, 2007; Al Houari *et al.*, 2008). To confirm the (E) conformation of the exocyclic C=C double bond, an X-ray crystal structure determination has been carried out. The present work is a continuation of the investigation of the dihydronaphthalene derivatives published recently by Akhazzane *et al.*, 2010.

The molecule of the title compound is formed by two fused rings linked to an ethyl group and to a 4-methoxybenzylidene moities as shown in Fig.1. The cyclohexanone ring adopts a screw-boat conformation as indicated by the total puckering amplitude QT = 0.477 (2) Å and spherical polar angle θ = 115.9 (2)° with φ = 35.6 (2)°. The benzene rings form a dihedral angle of 42.20 (8)°.

### S2. Experimental

The synthesis of the title compound was achieved using the method reported by Kerbal *et al.*, 1988. By a condensation of *para* anisaldehyde with 4-ethyl-3,4-dihydronaphthalen- 1(2*H*)-one in an alkaline medium in ethanol. The resulting residue was recrystallized from ethanol solution by slow evaporation to afford the title compound as colourless needles.

### S3. Refinement

H atoms were located in a difference map and treated as riding with C–H = 0.96 Å, C–H = 0.97 Å, and C–H = 0.93 Å for methyl, methylene and aromatic, respectively. All hydrogen with *U*_iso_(H) = 1.2 *U*_eq_ for methylene, aromatic and *U*_iso_(H) = 1.5 *U*_eq_ for methyl.

### Crystal data

**Table:**

C_20_H_20_O_2_	*D*_x_ = 1.234 Mg m^−^^3^
*M**_r_* = 292.36	Melting point: 383 K
Monoclinic, *P*2_1_/*c*	Mo *K*α radiation, λ = 0.71073 Å
*a* = 12.0411 (13) Å	Cell parameters from 4068 reflections
*b* = 8.9698 (9) Å	θ = 2.7–28.7°
*c* = 15.5832 (18) Å	µ = 0.08 mm^−^^1^
β = 110.721 (3)°	*T* = 296 K
*V* = 1574.2 (3) Å^3^	Needles, colourless
*Z* = 4	0.38 × 0.16 × 0.12 mm
*F*(000) = 624	

### Data collection

**Table:**

Bruker X8 APEX CCD diffractometer	2552 reflections with *I* > 2σ(*I*)
Radiation source: fine-focus sealed tube	*R*_int_ = 0.046
Graphite monochromator	θ_max_ = 28.7°, θ_min_ = 2.7°
φ and ω scans	*h* = −14→16
25378 measured reflections	*k* = −12→12
4068 independent reflections	*l* = −20→21

### Refinement

**Table:**

Refinement on *F*^2^	Hydrogen site location: inferred from neighbouring sites
Least-squares matrix: full	H-atom parameters constrained
*R*[*F*^2^ > 2σ(*F*^2^)] = 0.046	*w* = 1/[σ^2^(*F*_o_^2^) + (0.0546*P*)^2^ + 0.2497*P*] where *P* = (*F*_o_^2^ + 2*F*_c_^2^)/3
*wR*(*F*^2^) = 0.129	(Δ/σ)_max_ < 0.001
*S* = 1.01	Δρ_max_ = 0.16 e Å^−^^3^
4068 reflections	Δρ_min_ = −0.15 e Å^−^^3^
200 parameters	Extinction correction: *SHELXL2013* (Sheldrick, 2015), Fc^*^=kFc[1+0.001xFc^2^λ^3^/sin(2θ)]^-1/4^
0 restraints	Extinction coefficient: 0.0088 (16)

### Special details

**Table:**

Geometry. All e.s.d.'s (except the e.s.d. in the dihedral angle between two l.s. planes) are estimated using the full covariance matrix. The cell e.s.d.'s are taken into account individually in the estimation of e.s.d.'s in distances, angles and torsion angles; correlations between e.s.d.'s in cell parameters are only used when they are defined by crystal symmetry. An approximate (isotropic) treatment of cell e.s.d.'s is used for estimating e.s.d.'s involving l.s. planes.

### Fractional atomic coordinates and isotropic or equivalent isotropic displacement parameters (Å^2^)

**Table:**

	*x*	*y*	*z*	*U*_iso_*/*U*_eq_	
C1	0.64653 (13)	0.60708 (16)	0.83826 (9)	0.0452 (3)	
C2	0.52292 (13)	0.64122 (14)	0.83030 (9)	0.0415 (3)	
C3	0.49026 (15)	0.62928 (17)	0.90761 (10)	0.0520 (4)	
H3	0.5468	0.6017	0.9636	0.062*	
C4	0.37607 (17)	0.65769 (19)	0.90212 (11)	0.0614 (4)	
H4	0.3552	0.6486	0.9539	0.074*	
C5	0.29205 (16)	0.6999 (2)	0.81910 (12)	0.0621 (4)	
H5	0.2144	0.7191	0.8150	0.075*	
C6	0.32321 (14)	0.71366 (17)	0.74230 (10)	0.0514 (4)	
H6	0.2662	0.7429	0.6870	0.062*	
C7	0.43793 (12)	0.68471 (14)	0.74609 (9)	0.0405 (3)	
C8	0.47260 (12)	0.69755 (15)	0.66233 (9)	0.0415 (3)	
H8	0.4205	0.7717	0.6213	0.050*	
C9	0.60029 (13)	0.75335 (16)	0.68939 (10)	0.0466 (4)	
H9A	0.6231	0.7542	0.6356	0.056*	
H9B	0.6047	0.8549	0.7118	0.056*	
C10	0.68618 (12)	0.65723 (15)	0.76252 (9)	0.0432 (3)	
C11	0.79269 (12)	0.60980 (17)	0.76459 (10)	0.0485 (4)	
H11	0.8315	0.5479	0.8140	0.058*	
C12	0.85911 (12)	0.63703 (16)	0.70394 (10)	0.0464 (3)	
C13	0.84423 (13)	0.75855 (17)	0.64532 (11)	0.0521 (4)	
H13	0.7884	0.8310	0.6440	0.063*	
C14	0.91024 (13)	0.77445 (17)	0.58897 (11)	0.0530 (4)	
H14	0.8978	0.8561	0.5499	0.064*	
C15	0.99443 (13)	0.66918 (18)	0.59086 (11)	0.0525 (4)	
C16	1.01364 (15)	0.5491 (2)	0.65050 (12)	0.0643 (5)	
H16	1.0715	0.4788	0.6531	0.077*	
C17	0.94721 (14)	0.53437 (19)	0.70550 (12)	0.0602 (4)	
H17	0.9612	0.4534	0.7452	0.072*	
C18	0.45730 (13)	0.55031 (17)	0.60935 (10)	0.0509 (4)	
H18A	0.4894	0.5627	0.5608	0.061*	
H18B	0.5042	0.4744	0.6508	0.061*	
C19	0.33144 (15)	0.4944 (2)	0.56723 (12)	0.0692 (5)	
H19A	0.3303	0.4052	0.5329	0.104*	
H19C	0.2833	0.5692	0.5271	0.104*	
H19B	0.3006	0.4732	0.6149	0.104*	
O2	1.06414 (11)	0.67254 (15)	0.53835 (9)	0.0708 (4)	
C20	1.05297 (19)	0.7950 (2)	0.47844 (14)	0.0811 (6)	
H20A	1.1071	0.7828	0.4462	0.122*	
H20B	1.0711	0.8857	0.5134	0.122*	
H20C	0.9731	0.7995	0.4352	0.122*	
O1	0.71294 (10)	0.54013 (13)	0.90568 (7)	0.0630 (3)	

### Atomic displacement parameters (Å^2^)

**Table:**

	*U*^11^	*U*^22^	*U*^33^	*U*^12^	*U*^13^	*U*^23^
C1	0.0520 (8)	0.0409 (7)	0.0352 (7)	−0.0027 (6)	0.0061 (6)	0.0005 (6)
C2	0.0554 (8)	0.0349 (7)	0.0328 (7)	−0.0029 (6)	0.0138 (6)	−0.0014 (5)
C3	0.0705 (11)	0.0487 (8)	0.0354 (7)	−0.0050 (7)	0.0167 (7)	−0.0013 (6)
C4	0.0812 (12)	0.0662 (11)	0.0471 (9)	−0.0006 (9)	0.0356 (9)	−0.0030 (8)
C5	0.0641 (10)	0.0721 (11)	0.0578 (10)	0.0081 (8)	0.0311 (9)	−0.0022 (9)
C6	0.0540 (9)	0.0578 (9)	0.0428 (8)	0.0075 (7)	0.0179 (7)	0.0000 (7)
C7	0.0511 (8)	0.0347 (7)	0.0354 (7)	0.0002 (6)	0.0150 (6)	−0.0028 (6)
C8	0.0475 (8)	0.0427 (7)	0.0324 (7)	0.0081 (6)	0.0119 (6)	0.0052 (6)
C9	0.0515 (8)	0.0464 (8)	0.0421 (8)	0.0026 (6)	0.0170 (7)	0.0080 (6)
C10	0.0461 (8)	0.0407 (7)	0.0377 (7)	−0.0043 (6)	0.0086 (6)	−0.0005 (6)
C11	0.0450 (8)	0.0477 (8)	0.0435 (8)	−0.0032 (6)	0.0043 (6)	0.0020 (6)
C12	0.0367 (7)	0.0495 (8)	0.0461 (8)	−0.0051 (6)	0.0060 (6)	−0.0018 (7)
C13	0.0423 (8)	0.0444 (8)	0.0663 (10)	−0.0017 (6)	0.0151 (7)	0.0018 (7)
C14	0.0443 (8)	0.0502 (9)	0.0592 (9)	−0.0053 (7)	0.0119 (7)	0.0068 (7)
C15	0.0413 (8)	0.0611 (9)	0.0507 (9)	−0.0044 (7)	0.0110 (7)	−0.0033 (7)
C16	0.0520 (10)	0.0674 (11)	0.0740 (11)	0.0157 (8)	0.0227 (9)	0.0117 (9)
C17	0.0490 (9)	0.0637 (10)	0.0617 (10)	0.0096 (7)	0.0120 (8)	0.0167 (8)
C18	0.0550 (9)	0.0558 (9)	0.0396 (8)	0.0073 (7)	0.0138 (7)	−0.0057 (7)
C19	0.0635 (11)	0.0683 (11)	0.0614 (10)	0.0017 (9)	0.0042 (9)	−0.0173 (9)
O2	0.0642 (7)	0.0829 (9)	0.0717 (8)	0.0064 (6)	0.0320 (6)	0.0094 (7)
C20	0.0813 (14)	0.0945 (15)	0.0748 (13)	−0.0010 (11)	0.0367 (11)	0.0165 (11)
O1	0.0627 (7)	0.0745 (8)	0.0423 (6)	0.0079 (6)	0.0068 (5)	0.0166 (5)

### Geometric parameters (Å, º)

**Table:**

C1—O1	1.2285 (16)	C11—H11	0.9300
C1—C2	1.481 (2)	C12—C13	1.392 (2)
C1—C10	1.491 (2)	C12—C17	1.398 (2)
C2—C3	1.3975 (19)	C13—C14	1.385 (2)
C2—C7	1.4040 (19)	C13—H13	0.9300
C3—C4	1.371 (2)	C14—C15	1.378 (2)
C3—H3	0.9300	C14—H14	0.9300
C4—C5	1.384 (2)	C15—O2	1.3644 (19)
C4—H4	0.9300	C15—C16	1.387 (2)
C5—C6	1.381 (2)	C16—C17	1.370 (2)
C5—H5	0.9300	C16—H16	0.9300
C6—C7	1.386 (2)	C17—H17	0.9300
C6—H6	0.9300	C18—C19	1.508 (2)
C7—C8	1.5089 (18)	C18—H18A	0.9700
C8—C9	1.528 (2)	C18—H18B	0.9700
C8—C18	1.5337 (19)	C19—H19A	0.9600
C8—H8	0.9800	C19—H19C	0.9600
C9—C10	1.5092 (19)	C19—H19B	0.9600
C9—H9A	0.9700	O2—C20	1.417 (2)
C9—H9B	0.9700	C20—H20A	0.9600
C10—C11	1.341 (2)	C20—H20B	0.9600
C11—C12	1.459 (2)	C20—H20C	0.9600
			
O1—C1—C2	120.23 (13)	C12—C11—H11	114.0
O1—C1—C10	122.05 (14)	C13—C12—C17	116.60 (14)
C2—C1—C10	117.72 (12)	C13—C12—C11	125.69 (14)
C3—C2—C7	119.51 (14)	C17—C12—C11	117.70 (14)
C3—C2—C1	119.53 (13)	C14—C13—C12	121.82 (15)
C7—C2—C1	120.95 (12)	C14—C13—H13	119.1
C4—C3—C2	120.93 (14)	C12—C13—H13	119.1
C4—C3—H3	119.5	C15—C14—C13	119.92 (15)
C2—C3—H3	119.5	C15—C14—H14	120.0
C3—C4—C5	119.65 (15)	C13—C14—H14	120.0
C3—C4—H4	120.2	O2—C15—C14	125.17 (15)
C5—C4—H4	120.2	O2—C15—C16	115.27 (15)
C6—C5—C4	120.12 (16)	C14—C15—C16	119.56 (15)
C6—C5—H5	119.9	C17—C16—C15	119.89 (15)
C4—C5—H5	119.9	C17—C16—H16	120.1
C5—C6—C7	121.25 (15)	C15—C16—H16	120.1
C5—C6—H6	119.4	C16—C17—C12	122.17 (15)
C7—C6—H6	119.4	C16—C17—H17	118.9
C6—C7—C2	118.54 (13)	C12—C17—H17	118.9
C6—C7—C8	121.70 (12)	C19—C18—C8	115.56 (13)
C2—C7—C8	119.76 (13)	C19—C18—H18A	108.4
C7—C8—C9	110.23 (11)	C8—C18—H18A	108.4
C7—C8—C18	112.49 (12)	C19—C18—H18B	108.4
C9—C8—C18	110.38 (11)	C8—C18—H18B	108.4
C7—C8—H8	107.9	H18A—C18—H18B	107.5
C9—C8—H8	107.9	C18—C19—H19A	109.5
C18—C8—H8	107.9	C18—C19—H19C	109.5
C10—C9—C8	112.04 (11)	H19A—C19—H19C	109.5
C10—C9—H9A	109.2	C18—C19—H19B	109.5
C8—C9—H9A	109.2	H19A—C19—H19B	109.5
C10—C9—H9B	109.2	H19C—C19—H19B	109.5
C8—C9—H9B	109.2	C15—O2—C20	118.51 (14)
H9A—C9—H9B	107.9	O2—C20—H20A	109.5
C11—C10—C1	117.18 (13)	O2—C20—H20B	109.5
C11—C10—C9	126.41 (13)	H20A—C20—H20B	109.5
C1—C10—C9	116.38 (12)	O2—C20—H20C	109.5
C10—C11—C12	132.10 (14)	H20A—C20—H20C	109.5
C10—C11—H11	114.0	H20B—C20—H20C	109.5

## References

[bb1] Akhazzane, M., Zouihri, H., Daran, J.-C., Kerbal, A. & Al Houari, G. (2010). *Acta Cryst.* E**66**, o3067. 10.1107/S1600536810044387 PMC3011461 21589377

[bb2] Al Houari, G., Kerbal, A., Bennani, B., Baba, M. F., Daoudi, M. & Ben Hadda, T. (2008). *ARKIVOC*, **xii**, 42–50.

[bb3] Bennani, B., Kerbal, A., Daoudi, M., Filali Baba, B., Al Houari, G., Jalbout, A. F., Mimouni, M., Benazza, M., Demailly, G., Akkurt, M., Öztürk Yildirim, S. & Ben Hadda, T. (2007). *ARKIVOC*, **xvi**, 19–40.

[bb4] Bruker (2009). *APEX2*, *SAINT* and *SADABS*. Bruker AXS Inc., Madison, Wisconsin, USA.

[bb5] Burnett, M. N. & Johnson, C. K. (1996). *ORTEPIII*. Report ORNL-6895. Oak Ridge National Laboratory, Tennessee, USA.

[bb6] Farrugia, L. J. (2012). *J. Appl. Cryst.* **45**, 849–854.

[bb7] Kerbal, A., Tshiamala, K., Vebrel, J. & Laude, B. (1988). *Bull. Soc. Chim. Belg.* **97**, 149–161.

[bb8] Sheldrick, G. M. (2008). *Acta Cryst.* A**64**, 112–122. 10.1107/S0108767307043930 18156677

[bb9] Sheldrick, G. M. (2015). *Acta Cryst.* C**71**, 3–8.

[bb10] Spek, A. L. (2009). *Acta Cryst.* D**65**, 148–155. 10.1107/S090744490804362X PMC2631630 19171970

[bb11] Westrip, S. P. (2010). *J. Appl. Cryst.* **43**, 920–925.

