# Supplementary figures and images for: Crystal structure of (E)-4-ethyl-2-(4-meth­oxy­benzyl­idene)-3,4-di­hydro­naphthalen-1(2H)-one
Source: Acta Crystallogr E Crystallogr Commun. 2015 May 30;71(Pt 6):o432. doi: 10.1107/S205698901501004X (PMC4459325; doi:10.1107/S205698901501004X)

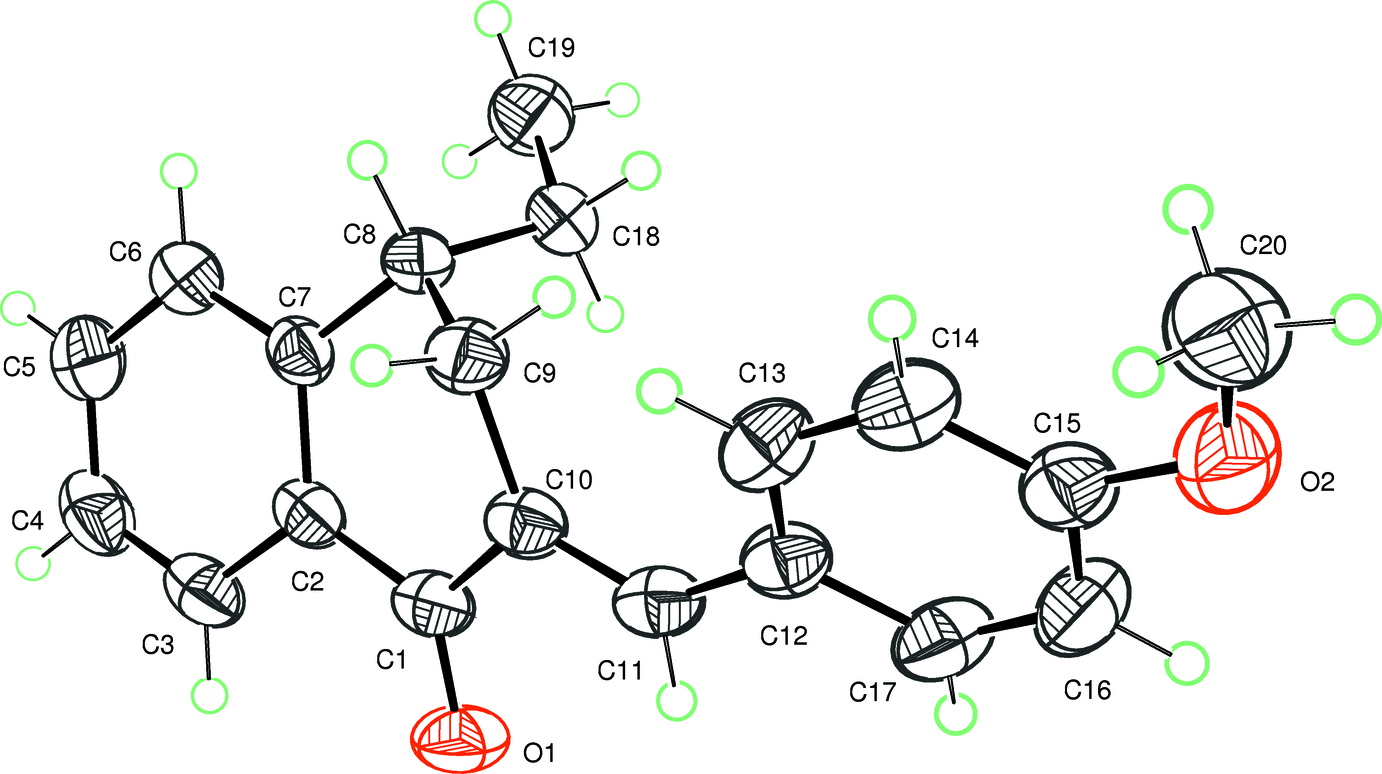

Supplement: Supplementary file 4 [file e-71-0o432-fig1.tif]
